# Protein Aggregation Inhibitors as Disease-Modifying Therapies for Polyglutamine Diseases

**DOI:** 10.3389/fnins.2021.621996

**Published:** 2021-02-12

**Authors:** Eiko N. Minakawa, Yoshitaka Nagai

**Affiliations:** ^1^Department of Degenerative Neurological Diseases, National Institute of Neuroscience, National Center of Neurology and Psychiatry, Kodaira, Japan; ^2^Department of Neurotherapeutics, Osaka University Graduate School of Medicine, Suita, Japan

**Keywords:** polyglutamine diseases, neurodegenerative diseases, aggregation inhibitor, protein misfolding, disease-modifying therapy, arginine

## Abstract

The polyglutamine (polyQ) diseases are a group of inherited neurodegenerative diseases caused by the abnormal expansion of a CAG trinucleotide repeat that are translated into an expanded polyQ stretch in the disease-causative proteins. The expanded polyQ stretch itself plays a critical disease-causative role in the pathomechanisms underlying polyQ diseases. Notably, the expanded polyQ stretch undergoes a conformational transition from the native monomer into the β-sheet-rich monomer, followed by the formation of soluble oligomers and then insoluble aggregates with amyloid fibrillar structures. The intermediate soluble species including the β-sheet-rich monomer and oligomers exhibit substantial neurotoxicity. Therefore, protein conformation stabilization and aggregation inhibition that target the upstream of the insoluble aggregate formation would be a promising approach toward the development of disease-modifying therapies for polyQ diseases. PolyQ aggregation inhibitors of different chemical categories, such as intrabodies, peptides, and small chemical compounds, have been identified through intensive screening methods. Among them, recent advances in the brain delivery methods of several peptides and the screening of small chemical compounds have brought them closer to clinical utility. Notably, the recent discovery of arginine as a potent conformation stabilizer and aggregation inhibitor of polyQ proteins both *in vitro* and *in vivo* have paved way to the clinical trial for the patients with polyQ diseases. Meanwhile, expression reduction of expanded polyQ proteins *per se* would be another promising approach toward disease modification of polyQ diseases. Gene silencing, especially by antisense oligonucleotides (ASOs), have succeeded in reducing the expression of polyQ proteins in the animal models of various polyQ diseases by targeting the aberrant mRNA with expanded CAG repeats. Of note, some of these ASOs have recently been translated into clinical trials. Here we overview and discuss these recent advances toward the development of disease modifying therapies for polyQ diseases. We envision that combination therapies using aggregation inhibitors and gene silencing would meet the needs of the patients with polyQ diseases and their caregivers in the near future to delay or prevent the onset and progression of these currently intractable diseases.

## Introduction

The polyglutamine (polyQ) diseases are a group of inherited neurodegenerative diseases that are caused by the abnormal expansion of a CAG triplet repeat (above 35–40 repeats) in the coding region within the causative gene of each disease. This expanded CAG repeat is translated into an expanded polyQ stretch in the resultant protein product (Orr and Zoghbi, 2007). At least nine diseases including spinocerebellar ataxia (SCA) types 1, 2, 3, 6, 7, and 17, Huntington’s disease (HD), spinal and bulbar muscular atrophy (SBMA), and dentatorubral pallidoluysian atrophy (DRPLA) are known so far to belong to this group of diseases (Table 1; Stoyas and La Spada, 2018).

**TABLE 1:** The polyglutamine diseases.

Disease	Gene	CAG repeat length	
		Normal	Disease
Spinal and bulbar muscular atrophy (SBMA)	Androgen receptor (AR)	9–36	38–65
Huntington’s disease (HD)	Huntingtin (HTT)	6–35	36–180
Spinocerebeller ataxia type 1 (SCA1)	Ataxin 1 (ATXN1)	6–39	39–83
Spinocerebeller ataxia type 2 (SCA2)	Ataxin 2 (ATXN2)	14–32	32–200
Spinocerebeller ataxia type 3 (SCA3)	Ataxin 3 (ATXN3)	12–41	55–84
Spinocerebeller ataxia type 6 (SCA6)	Calcium voltage-gated channel subunit alpha1 A (CACNA1A)	4–19	20–33
Spinocerebeller ataxia type 7 (SCA7)	Ataxin 7 (ATXN7)	4–35	37–306
Spinocerebeller ataxia type 17 (SCA17)	TATA-box binding protein (TBP)	25–44	46–63
Dentatorubral pallidoluysian atrophy (DRPLA)	Atrophin 1 (ATN1)	6–36	49–88

The pathological hallmarks of polyQ diseases are the inclusion bodies that mainly consist of proteins with an expanded polyQ stretch and the progressive neuronal cell loss in the regions within the brains or spinal cords that are specific to each disease (Takeuchi and Nagai, 2017). Patients suffer from a variety of motor, cognitive, and psychiatric impairments that depends on the regions affected in the nervous system in each disease. Disease-modifying treatments that delay or halt the onset or progression of polyQ diseases remain an unmet clinical need (Nagai and Minakawa, 2015).

The causative genes of the nine polyQ diseases have neither sequence homology nor any functional similarities, except for the expanded CAG repeat that encodes an expanded polyQ stretch (Paulson, 2018). A wide variety of the cellular and molecular pathogenic events that are induced by these proteins with expanded polyQ stretch are largely shared by the nine different polyQ diseases (Stoyas and La Spada, 2018). Various studies using invertebrate and vertebrate animal models of polyQ diseases have shown that expanded polyQ stretch itself is sufficient to induce neuronal degeneration and leads to neurological impairment *in vivo* (Burright et al., 1995; Ikeda et al., 1996; Warrick et al., 1998; Faber et al., 1999). These findings indicate the critical disease-causative role of the expanded polyQ stretch in the pathogenesis of polyQ diseases.

**FIGURE 1:**
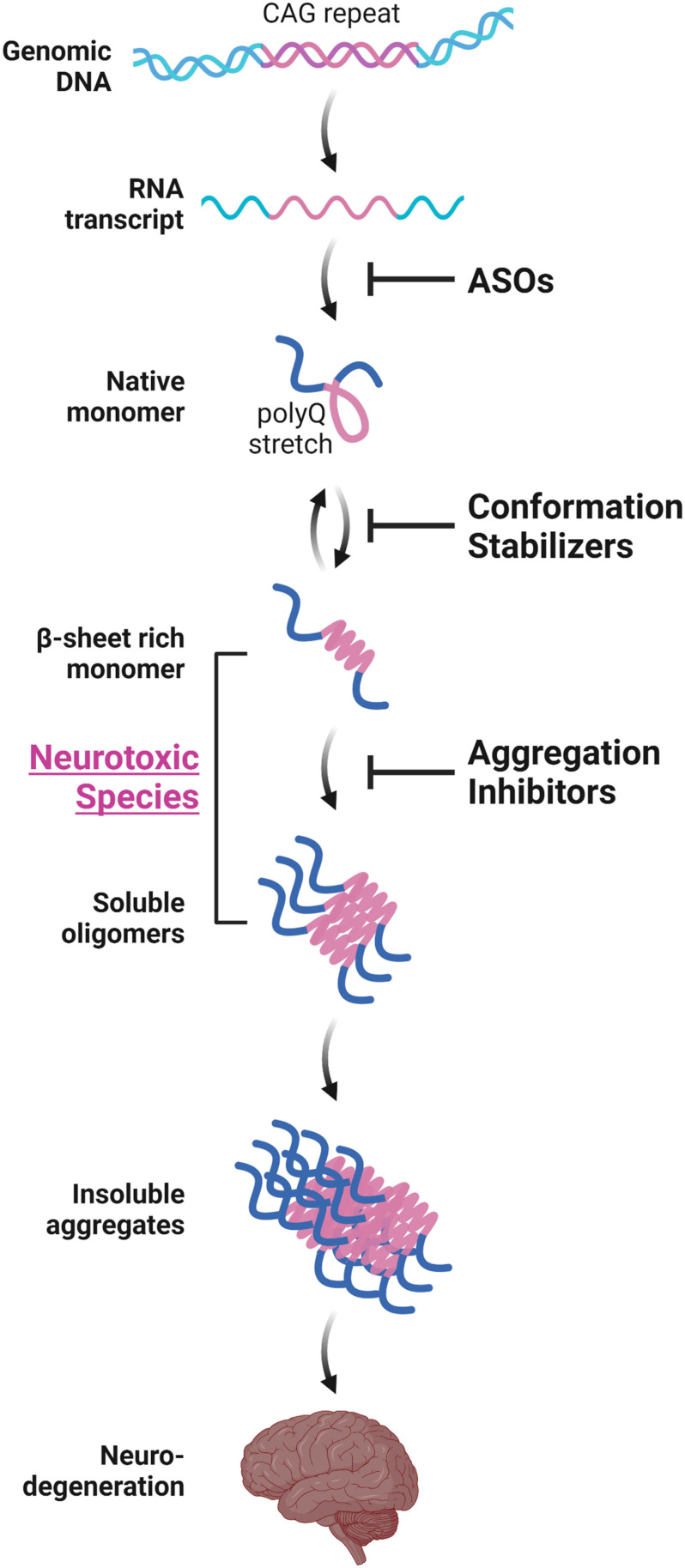
Aggregation-process-specific therapeutic targets of the polyQ diseases. Expanded CAG repeats in the disease-causative genes of polyQ diseases produce disease-causing proteins with an expanded polyQ stretch. Such proteins undergo a conformation transition from an α-helix-rich structure into the β-sheet-rich structure at a monomeric state, followed by the formation of soluble oligomers and then insoluble aggregates with amyloid fibrillar structures, eventually leading to neurodegeneration. Conformation stabilizers and aggregation inhibitors are strong candidate molecules for the disease-modifying therapies for polyQ diseases. These therapeutic molecules can be used in combination with antisense oligonucleotides (ASOs) that reduces the expression of polyQ proteins.

Various *in vitro* structural studies including ours have shown that the expanded polyQ stretch undergoes a conformational transition from the native monomer into the β-sheet-rich monomer, followed by the formation of soluble oligomers and then insoluble aggregates with amyloid fibrillar structures (Figure 1; Chen et al., 2001, 2002; Masino et al., 2002; Poirier et al., 2002; Khare et al., 2005; Nagai et al., 2007; Nucifora et al., 2012). The presence of these intermediate soluble species preceding the formation of insoluble aggregates was confirmed *in vivo* using cultured cells (Takahashi et al., 2007, 2008; Olshina et al., 2010) and the brains of the HD (Legleiter et al., 2010; Sathasivam et al., 2010) and SBMA (Li et al., 2007) mice models. The presence of polyQ proteins with oligomer-like structure was also confirmed in the brains of patients with HD (Legleiter et al., 2010). Importantly, these intermediate soluble species exhibit significant neuronal toxicity (Kayed et al., 2003; Miller et al., 2011). Of note, we demonstrated that the monomeric conformer of the expanded polyQ protein with β-sheet-rich structure, as well as oligomers, exhibit cellular toxicity (Figure 1; Nagai et al., 2007). In contrast, α-helical coiled-coil structure also has been demonstrated to contribute, at least in part, to the toxicity of polyQ proteins (Fiumara et al., 2010; Kwon et al., 2018). Of note, Fiumara et al. (2010) demonstrated that polyQ peptides themselves form α-helical coiled-coil structure and assemble into oligomers, and those mutations that enhance coiled-coil propensity of polyQ proteins lead to increased aggregation and toxicity in cultured cells. The α-helical coiled-coil structure of polyQ proteins were also prominently involved in protein-protein interaction (Fiumara et al., 2010). In line with these findings, quantitative proteome of the insoluble fraction in HD mice model revealed that proteins sequestered with the polyQ aggregates were enriched with those containing coiled-coil structures (Hosp et al., 2017). In either case, formation of polyQ-positive aggregates or inclusion bodies *per se* does not correlate with neuronal cell death, and even decreases the risk of neuronal cell death, and hence may be a protective response of the cells against the intermediate soluble but toxic polyQ protein species (Klement et al., 1998; Saudou et al., 1998; Kuemmerle et al., 1999; Arrasate et al., 2004; Kim et al., 2016). Taken altogether, these findings indicate the significance of protein conformation stabilization and aggregation inhibition of the intermediate soluble species of expanded polyQ proteins toward the development of disease-modifying therapies for polyQ diseases (Figure 1; Takeuchi and Nagai, 2017).

## Disease-Modifying Therapies for Polyglutamine Diseases *via* Targeting Conformation Transition and Aggregation of Expanded Polyglutamine Proteins

To achieve disease modification for polyQ diseases *via* conformation stabilization and aggregation inhibition of polyQ proteins, molecules of various categories have been screened using different approaches. Initial studies mainly searched for molecules such as intrabodies or peptides that directly bind to the expanded polyQ stretch. Such molecules are expected to exhibit anti-aggregation effect through alternation of the protein folding process because the protein folding kinetics are sensitive to the residues surrounding the protein itself (Robertson et al., 2011; Wetzel, 2012). In addition, such molecules may exhibit therapeutic effects by affecting the turnover or subcellular localization of the expanded polyQ proteins (Messer and Butler, 2020). On the other hand, various molecules including small chemical compounds have been screened using *in vitro* assays that directly test their anti-aggregation property. Molecules that were identified through such screenings are expected to exhibit therapeutic effect through direct or indirect molecular interaction with expanded polyQ proteins as discussed below.

### Intrabodies

Intrabody is an antibody fragment that is bioactive inside cells and binds specifically to intracellular antigens (Messer and Butler, 2020). Since the identification of the first single-chain Fv (scFv) antibody, scFvC4, that specifically binds to the N-terminal region of HTT (Lecerf et al., 2001) and suppresses the formation of mutant HTT (mHTT)-positive aggregates *in vivo* (Wolfgang et al., 2005; Snyder-Keller et al., 2010), several intrabodies that inhibits mHTT aggregation and ameliorates the behavioral phenotypes of various HD animal models have been identified (Wang et al., 2008; Southwell et al., 2009, 2011; Amaro and Henderson, 2016).

The advantage of intrabodies for the treatment of HD is their high binding affinity to HTT protein, which could alter the misfolding process of mHTT protein as well as affect the turnover or subcellular localization of mHTT protein. On the contrary, when mHTT protein becomes fibrillar and insoluble, it can no longer be corrected by the intrabodies that have been developed so far (Messer and Butler, 2020). Another concern is that neither of the currently available intrabodies for HD do not exhibit long-term effect. In addition, the administration route of intrabodies to the brain is currently limited to gene delivery *via* viral vectors due to the large molecular size of intrabodies. Overcoming these limitations would bring intrabodies closer to clinical utility.

### Peptides and Small Chemical Compounds

As discussed above, one of the critical issues to develop disease-modifying treatments for polyQ diseases is to enable repetitive and long-term delivery of the drugs to the brain. To overcome this issue, extensive screenings for low molecular weight compounds such as peptides and small chemical compounds that inhibit the aggregation of expanded polyQ proteins have been performed. So far, various peptides and small chemical compounds have been proven to exhibit anti-aggregation propensity against expanded polyQ proteins through direct or indirect molecular interaction with expanded polyQ proteins. Among them, a number of peptides or small chemical compounds have been demonstrated to exhibit neuroprotective effect in various *in vivo* models of polyQ diseases, as will be discussed in the following sections. As an alternative approach, various peptides and chemical compounds that exert therapeutic effects for polyQ disease models by activating the cellular protective mechanisms against expanded polyQ proteins, such as molecular chaperones, ubiquitin-proteasome system, and autophagy, have also been developed. In addition, several peptides and chemical compounds have also been developed that inhibit the downstream events such as aberrant calcium signaling or apoptosis evoked by expanded polyQ proteins and lead to cellular dysfunction or cell death. Peptides and chemical compounds targeting molecular processes other than the aggregation of polyQ proteins are beyond the scope of this article and have been recently reviewed elsewhere (Takeuchi et al., 2014; Takeuchi and Nagai, 2017).

#### Peptides

Peptides that specifically bind to expanded polyQ proteins but not to polyQ proteins with non-pathogenic length of polyQ stretch have been screened as aggregation inhibitor candidates of expanded polyQ proteins. Peptides that recognize and bind to this specific conformation of expanded polyQ proteins were expected to alter the kinetics of the protein misfolding and slow down or prevent the aggregation process, as was the case for intrabodies. This strategy was in accord with the identification of anti-polyQ monoclonal antibody1C2 that preferentially binds to polyQ proteins with longer polyQ stretch (Trottier et al., 1995), which implies the length-dependent difference in the tertiary structure of polyQ proteins.

We previously screened for peptides that selectively bind to the expanded polyQ stretch using a combinatorial peptide phage display libraries, and successfully identified several polyQ-binding peptides including QBP1 (polyQ binding peptide 1; SNWKWWPGIFD) (Nagai et al., 2000). QBP1 exhibited a preferential high affinity for the expanded polyQ stretch with a dissociation constant (Kd) of 5.7 μM (Okamoto et al., 2009). QBP1 inhibits the toxic β-sheet conformation transition of the polyQ protein monomer and its subsequent aggregation *in vitro* (Nagai et al., 2007). Further studies on its structure–activity relationship identified that the tryptophan-rich sequence is indispensable for the anti-aggregation property of QBP1 (Ren et al., 2001; Hamuro et al., 2007; Tomita et al., 2009) and that the original 11 amino acid sequence of QBP1 can be shortened to eight amino acids (WKWWPGIF) without losing its anti-aggregation property against polyQ proteins. QBP1 expression in cultured cells (Nagai et al., 2000, 2007; Takahashi et al., 2007, 2008) or in *Drosophila* models of polyQ diseases (Nagai et al., 2003) significantly suppressed the formation of polyQ inclusions and polyQ-induced cell death. We also conjugated QBP1 with protein transduction domains (PTD-QBP1) to increase its membrane permeability, and showed that intracellular delivery of PTD-QBP1 successfully suppressed polyQ-induced premature death in *Drosophila* by oral administration of PTD-QBP1 (Popiel et al., 2007). Although we further examined the effect of PTD-QBP1 on polyQ disease model mice, repeated intraperitoneal injection of PTD-QBP1 modestly improved only the body weight loss but not the motor phenotypes nor the inclusion formation in the brains (Popiel et al., 2009), probably due to insufficient delivery across the blood-brain barrier.

Recently, brain delivery methods of QBP1 were investigated. Intranasal administration of QBP1 using a thermosensitive gel with chitosan, an absorption enhancer for nasal delivery (Illum et al., 2001), was demonstrated to significantly elevate the concentration of QBP1 in rodents’ brains for up to 10-fold compared with intravenous administration or intranasal administration of water-dissolved QBP1 (Yang et al., 2018). In another recent study, biodegradable poly-_*D,L*_-lactide-*co*-glycolide (PLGA) nanoparticles encapsulating QBP1 was shown to inhibit polyQ protein aggregation in cultured neuronal cells and to suppress the motor dysfunction of *Drosophila* model of polyQ disease (Joshi et al., 2019). Considering that QBP1 was recently found to bind and exert anti-aggregation property not only to polyQ proteins but also to glutamine-rich regions of the pathogenic amyloidogenic proteins such as TDP-43 (Mompeán et al., 2019), an aggregation-prone protein involved in the pathomechanisms of amyotrophic lateral sclerosis (ALS)/frontotemporal dementia (FTD), further studies are strongly awaited to increase the bioavailability of QBP1 itself or its derivatives or analogs.

Chen et al. (2011) also screened for potential polyQ aggregation inhibitors by a combinatorial screening for a library consisting of peptoids, a class of peptidomimetics comprised of N-substituted glycine oligomers. Peptoids are resistant to degradation by protease and more permeable to cellular membranes compared with peptides (Simon et al., 1992). Through a screening of 60,000 diverse peptoids, they identified HQP09 as a specific ligand of expanded polyQ proteins (Chen et al., 2011). HQP09 suppressed polyQ aggregation *in vitro*, reduced polyQ-induced cell death in primary neuronal cultures, and decreased polyQ inclusion bodies in polyQ disease model mice by intracerebroventricular administration (Chen et al., 2011). Intriguingly, HQP09 and QBP1 did not compete with each other in binding expanded polyQ proteins, suggesting that HQP09 and QBP1 recognize the expanded polyQ stretch in non-overlapping sites or structures (Chen et al., 2011). They further identified critical residues for HQP09 activity and generated HQP09_9, a minimal derivative of HQP09, that maintains the specificity in polyQ-binding property and polyQ aggregation inhibition property *in vitro*, and neuroprotective effects in primary neuronal cultures derived from polyQ disease model mice (Chen et al., 2011). Although subcutaneous administration of HQP09_9 did not exhibit therapeutic effect on polyQ disease model mice, HQP09_9 would serve as a lead compound to generate a novel polyQ aggregation inhibitor with better bioavailability.

Besides these screening approaches, recent studies have succeeded in designing aggregation inhibitor peptides with bipartite structures that consist of a domain with the ability to interact with aggregation-prone proteins and a domain with the ability to prevent their aggregation. He et al. (2020) designed a bipartite peptide, 8R10Q that harbors a polyQ attached to a positively charged polyarginine. The polyQ sequence of 8R10Q was expected to exhibit a specific and high affinity to the polyQ stretch in mHtt protein through its self-aggregating property, while the polyarginine sequence was expected to enable 8R10Q to penetrate cell membrane, increase solubility and prevent self-aggregation of 8R10Q, and prevent the mHtt/8R10Q hybrid from self-aggregation. As expected, 8R10Q suppressed the oligomer and inclusion formation of mHTT proteins in cultured cells. In addition, intranasal administration of iodine-124-labeled 8R10Q resulted in delivery of 8RQ10 to the brains, suppression of motor deficit, prolonged survival, and amelioration of mHTT aggregation and neuronal death in HD mouse model.

#### Small Chemical Compounds

Although various intrabodies, peptides, or peptoids that suppress the toxic conformation transition and aggregation of expanded polyQ proteins have been identified as discussed above, they are still of limited clinical use mainly due to poor drug delivery into mammalian brains. Screening for small chemical compounds that possess anti-aggregation property against expanded polyQ proteins both *in vitro* and *in vivo*, especially in mammalians, have therefore been intensively performed.

Since the first report by Wanker and colleagues (Heiser et al., 2002), various groups including ours have performed high-throughput screenings for polyQ aggregate inhibitors from large-scale chemical compound libraries using *in vitro* systems (Heiser et al., 2002; Tanaka et al., 2004; Ehrnhoefer et al., 2006) or cellular systems (Pollitt et al., 2003; Zhang et al., 2005; Fuentealba et al., 2012), although most of them have limitations for clinical application due to their toxicity, poor BBB permeability, or metabolic instability (Hockly et al., 2006; Frid et al., 2007). Several compounds among them have successfully exerted therapeutic effects in various *in vivo* models of polyQ diseases. For example, (-)-epigallo-catechin-3-gallate (EGCG), a green tea polyphenol, ameliorates neurodegeneration in a *Drosophila* model of HD (Ehrnhoefer et al., 2006) and a *Caenorhabditis elegans* model of SCA3 (Bonanomi et al., 2014). Trehalose, a disaccharide, delays the onset of neurological symptoms in a mouse model of HD (Tanaka et al., 2004). Y-27632, a Rho-activated protein kinase (ROCK) inhibitor, suppresses neurodegeneration in a *Drosophila* (Pollitt et al., 2003) and mouse model of HD (Li et al., 2009). HA-1077, a clinically approved ROCK inhibitor, also suppresses the polyQ-induced retinal degeneration when administered intravitreally in HD mouse model (Li et al., 2013). C2-8, a sulfobenzoic acid derivative, ameliorates neurodegeneration in a *Drosophila* model of HD (Zhang et al., 2005), although its effect in mice remains controversial despite its BBB permeability (Chopra et al., 2007; Wang et al., 2013).

Some of the small chemical compounds that exert anti-aggregation property on other disease-causing proteins of neurodegenerative diseases, e.g., amyloid-β (Aβ), tau, prion, and α-synuclein (α-Syn), also inhibits aggregation of polyQ proteins *in vivo*. For example, N′-benzylidenebenzohydrazide derivatives, an aggregation inhibitor of prion protein, suppresses polyQ protein aggregation in a zebrafish model of HD (Schiffer et al., 2007). Methylene blue, an aggregation inhibitor of tau, also suppresses polyQ protein aggregation and neurological phenotypes of the *Drosophila* and mouse models of HD (Sontag et al., 2012). Curcumin, an aggregation inhibitor of Aβ, α-Syn, and prion protein, inhibits polyQ aggregation in yeast model (Verma et al., 2012) and knock-in mouse model (Hickey et al., 2012) of HD, although it did not ameliorate the motor deficit in the knock-in mouse model (Hickey et al., 2012). Intriguingly, a recent study showed that lipid membranes are a key modifier of the ability of small chemical compounds to inhibit polyQ aggregation (Beasley et al., 2019). While the polyQ aggregation inhibition property of curcumin was diminished under the presence of 1-palmitoyl-2-oleoyl-glycero-3-phosphocholine or total brain lipid extract vesicles, that of EGCG was not affected (Beasley et al., 2019). This study suggests the importance of taking into account the molecules surrounding the polyQ proteins in a crowded cellular environment in addition to the aggregation of polyQ protein *per se*.

Recently, we and others independently identified arginine as a potent aggregation inhibitor of polyQ proteins (Minakawa et al., 2020). Arginine belongs to a group of low molecular weight molecules named chemical chaperones. Chemical chaperones facilitate proper protein folding and suppress protein aggregation by stabilizing proteins in their native conformation and influencing the rate or fidelity of the protein-folding reaction (Welch and Brown, 1996; Cortez and Sim, 2014). Trehalose (Tanaka et al., 2004), proline (Ignatova and Gierasch, 2006), and cyclohexanol (McLaurin et al., 2006) are chemical chaperones that were previously shown to inhibit the aggregation of disease-causing proteins including the polyQ proteins. Meanwhile, arginine has been well known to exhibit non-specific anti-aggregation effect and is commonly used as an additive to prevent aggregation of various recombinant proteins expressed in *Escherichia coli* (Arakawa and Tsumoto, 2003). In addition, arginine has a high BBB permeability (Pardridge, 1983) and an established safety profile in humans since it is in clinical use for other human diseases such as urea cycle deficits and mitochondrial myopathy, encephalopathy, lactic acid, and stroke (MELAS) syndrome (Koga et al., 2002; Boenzi et al., 2012; Koenig et al., 2016). Although there are reports of diarrhea with varying incidence following oral administration of arginine especially at single high-dose administration possibly due to arginine-induced water and electrolyte secretion (Grimble, 2007), long-term oral administration of relatively high-dose arginine (0.3–0.5 g/kg/day orally in three divided doses after each meal for 2 years) is reported to be well tolerable with no severe adverse effect related with arginine (Koga et al., 2018).

We identified arginine as a potent polyQ aggregation inhibitor by screening a library of representative chemical chaperones using an *in vitro* polyQ aggregation assay (Minakawa et al., 2020). Importantly, arginine suppresses the most upstream process of polyQ protein aggregation, which is the toxic conformational transition of polyQ proteins from an α-helix-rich monomer to a toxic β-sheet-rich monomer, and also the downstream process such as the oligomer formation, aggregation formation, and seed-induced aggregation of polyQ proteins (Minakawa et al., 2020). Oral administration of arginine suppresses motor deficit, polyQ aggregation pathology, and neurodegeneration of multiple *in vivo* invertebrate and vertebrate models of polyQ diseases including two different mice models of SCA1 and SBMA (Minakawa et al., 2020). Of note, arginine exerted therapeutic effect on the motor deficit of SCA1 model mice even when administered after symptom onset (Minakawa et al., 2020). Based on these results and the established safety of arginine in humans, we have scheduled a clinical trial to evaluate the efficacy and safety of long-term arginine administration in polyQ patients in Japan.

In a recent independent study, Singh et al. (2019) showed that arginine and arginine ethyl ester (AEE) suppresses the aggregation of HTT exon 1 with an expanded polyQ stretch (mHTTex1) and rescues the motor deficit of the *Drosophila* HD model. Arginine consists of a guanidino group in the N-terminus and a glycine group in the C-terminus. They identified that the guanidino group of arginine is necessary for its anti-aggregation propensity against mHTTex1. Meanwhile, AEE and arginine methyl ester showed stronger anti-aggregation propensity than arginine (Singh et al., 2019). Arginine does not directly bind to mHTTex1, but instead alters the hydrogen bonding network (Singh et al., 2019). In contrast, AEE directly binds to mHTTex1 at the N-terminal domain in addition to altering the hydrogen bonding network (Singh et al., 2019). Consistently, AEE exhibited a higher anti-aggregation effect on mHTTex1, and a better rescue of the motor deficit of *Drosophila* HD model compared with arginine (Singh et al., 2019).

These recent two studies suggest that arginine is a promising candidate molecule for the disease-modifying therapy of polyQ diseases, although it should be noted that a slight but significant elevation of plasma arginine level is reported in an animal model of HD (Skene et al., 2017) and that it is not still conclusive whether this elevation is protective or pathogenic for HD. Meanwhile, the non-specific anti-aggregation property of arginine would enable the use of arginine for the treatment of neurodegenerative disease in common, since protein misfolding is the common pathomechanism underlying neurodegenerative diseases such as Alzheimer’s disease (AD) or Parkinson’s disease (PD). Indeed, arginine suppresses the aggregation of Aβ or α-Syn *in vitro* (Das et al., 2007; Ghosh et al., 2018), both of which aggregate, exhibit neurotoxicity, and accumulate in the brains of patients with AD or PD. Alternatively, arginine derivatives with modification in the glycine group but not the guanidino group would help increase the specificity and efficacy of arginine as a polyQ aggregation inhibitor. Meanwhile, as we previously discussed, formation of polyQ-positive aggregates may be a protective response of the cells against the intermediate soluble but toxic protein species. From this point of view, it should be noted that the aforementioned aggregation inhibitors, not limited to arginine and its derivatives, might have exhibited neuroprotective effects through their ability to modulate the toxicity of these proteins, e.g., by altering protein interaction (Kim et al., 2016), in addition to their *in vitro* ability to modulate the aggregation process.

## Disease-Modifying Therapies for Polyglutamine Diseases *via* Reduction of Toxic Polyglutamine Protein Expression Using Antisense Oligonucleotides

Besides the aggregation inhibition of polyQ proteins by various aforementioned methods, reducing the expression of expanded polyQ proteins *per se* is an attractive approach toward the development of disease-modification therapy for polyQ diseases. Among the preclinical studies using RNA interference (RNAi)-based methods or antisense oligonucleotides (ASOs), both of which alter the polyQ protein expression by targeting the aberrant mRNA with expanded CAG repeats, ASOs for the treatment of HD have recently been translated into clinical trials. In addition to the therapeutic effects of ASOs, the safety, tolerability, and effective delivery of ASOs in humans have been established in clinical studies for spinal muscular atrophy or Duchenne muscular dystrophy, and lead to the recent approval of the ASOs such as nusinersen or eteplirsen, respectively, by the Food and Drug Administration in the United States (Schoch and Miller, 2017). ASOs therefore are promising candidates for the disease-modifying therapies for polyQ diseases which could be used in combination with aggregation inhibitors (Figure 1).

Antisense oligonucleotides are synthetic single-stranded oligonucleotides that bind to RNA molecules. ASOs can be designed in an allele-specific or non-allele-specific manner to reduce the expression of target protein either by degrading the mRNA *via* recruitment of RNase H, inducing exon skipping by regulating the splicing machinery at specific exon-intron junctions, or inhibiting protein translation by interfering with the translational machinery (Bennett and Swayze, 2010). Initial preclinical studies utilizing ASOs for the treatment of polyQ diseases took allele-specific approaches and targeted either the expanded CAG repeat in the mRNA (Hu et al., 2009) or single nucleotide polymorphisms (SNPs) that are associated with the disease (Carroll et al., 2011). Allele-specific ASOs targeting the mutated gene would be favorable when the protein product from wild-type mRNA is physiologically indispensable. However, this approach might not be applicable to patients homozygous for disease-causing mutations or disease-related SNPs. In addition, especially in polyQ diseases, allele-specific ASOs targeting CAG repeats in the mutant allele may result in the reduced expression of physiologically indispensable proteins because the wild-type allele of the disease-causing genes and genes of various other proteins also contain CAG repeats, although chemical modification of ASOs may help overcome this limitation (Rué et al., 2016). Furthermore, multiple allele-specific ASOs for a single disease need to be developed when multiple SNPs are associated with the disease, as is the case with polyQ diseases. Accordingly, most preclinical studies have utilized non-allele specific ASOs or allele-specific ASOs for disease-related SNPs associated with polyQ diseases.

Non-allele specific ASO targeting *ATXN1* significantly reduced the expression of ATXN1, ameliorated the motor deficit, and prolonged the survival of SCA1 knock-in mice by intracerebroventricular (ICV) administration (Friedrich et al., 2018). Similarly, ICV administration of non-allele specific ASO targeting *ATXN2* significantly reduced the expression of ATXN2 and ameliorated the motor deficit in two different mice models of SCA2 (Scoles et al., 2017). Of note, this ASO restored the firing rate of Purkinje cells even when administered after the motor phenotype onset of the SCA2 knock-in mice (Scoles et al., 2017). ICV administration of non-allele specific ASO targeting *ATXN3* also significantly reduced the expression of ATXN3, ameliorated the motor deficit, and restored the firing rate of Purkinje cells (McLoughlin et al., 2018). Importantly, this ASO successfully reduced the high molecular weight ATXN3 aggregates and prevented the nuclear localization of ATXN3 in two different mice models of SCA3 (Moore et al., 2017; McLoughlin et al., 2018). Non-allele specific ASOs for SCA7 (Niu et al., 2018), HD (Kordasiewicz et al., 2012; Stanek et al., 2013), and SBMA (Lieberman et al., 2014; Sahashi et al., 2015), and allele-specific ASOs for HD (Sun et al., 2014; Rué et al., 2016; Datson et al., 2017; Southwell et al., 2018) that are effective in the mice or non-human-primate model of each disease have also been identified.

Based on these studies, the first phase 1/2 clinical trial was performed using a non-allele-specific ASO in patients with HD (Tabrizi et al., 2019). This ASO showed good tolerability without serious adverse effects, and induced a dose-dependent reduction of mHTT in the cerebrospinal fluid (Tabrizi et al., 2019). An open-labeled extension study for patients with HD who completed the phase 1/2 trial (NCT03342053) and a phase 3 trial (NCT03761849) using the same ASO is ongoing. In addition, two other phase 1/2 trials (NCT03225833 and NCT03225846) are also ongoing, which uses allele-specific ASOs that target mutant-allele-associated SNPs.

The advantages of ASOs in the treatment of polyQ diseases are their high specificity and established safety, which have led to successful translation of preclinical to clinical studies in HD as discussed above. Meanwhile, although ASOs apparently distributes widely throughout the brain by intrathecal or ICV administration in rodents and non-human primates, ASO concentration may be lower in deeper brain regions such as the striatum (Kordasiewicz et al., 2012). Although intrathecal delivery of ASOs showed therapeutic effects in the clinical trials for SMA, this might have been because SMA mainly affects the spinal cord. In addition, currently available ASOs needs repeated administration to maintain the therapeutic effect. Development of better ASO administration methods that ensure prolonged delivery to the target brain regions and are less invasive is strongly awaited. In addition, the extremely high cost of the FDA-approved ASOs remains a challenge in clinical settings (Burgart et al., 2018).

## Conclusion

Disease-modifying treatment for polyQ diseases have long been an unmet clinical need. Although various chemical compounds have been demonstrated to inhibit the aggregation of polyQ proteins *in vitro*, most of them were of limited use in the clinical settings due to their toxicity, poor BBB permeability, or metabolic instability. Among the aggregation inhibitors of polyQ proteins, recent studies have identified promising candidate therapeutic molecules that is effective *in vivo* and could achieve prompt clinical application. One of such molecules is arginine, which already has an established safety in humans and fits the recent concept of “drug repositioning” (Corbett et al., 2012). Notably, arginine was effective in polyQ disease model mice even after symptom onset. Meanwhile, great efforts are under way to achieve better drug delivery of potent aggregation inhibitor peptides, peptoids or intrabodies. Together with the recent success of ASOs in the clinical trials to reduce the expression of polyQ proteins in patients with HD, combination therapies using gene silencing and aggregation inhibitors for the remaining polyQ proteins would enhance the possibility for delaying or halting the onset and progression of polyQ diseases in the near future.

## Author Contributions

EM and YN designed the manuscript and assessed the literature. EM wrote the initial draft of the manuscript. EM and YN edited the subsequent drafts and revisions. Both authors contributed to the article and approved the submitted version.

## Conflict of Interest

YN belongs to an endowment department, supported by Nihon Medi-Physics Co., AbbVie GK., Otsuka Pharm Co., Kyowakai Med. Co., Fujiikai Med. Co., Yukioka Hosp., Osaka Gyoumeikan Hosp., Kyorin Co., and Tokuyukai Med. Co. The remaining author declares that the research was conducted in the absence of any commercial or financial relationships that could be construed as a potential conflict of interest.
